# Cytokine Mixtures Mimicking Secretomes From Mesenchymal Stem Cells Improve Medication‐Related Osteonecrosis of the Jaw in a Rat Model

**DOI:** 10.1002/jbm4.10013

**Published:** 2017-09-06

**Authors:** Kenichi Ogata, Mayu Matsumura, Masafumi Moriyama, Wataru Katagiri, Hideharu Hibi, Seiji Nakamura

**Affiliations:** ^1^ Section of Oral and Maxillofacial Oncology Division of Maxillofacial Diagnostic and Surgical Sciences Faculty of Dental Science Kyushu University Fukuoka Japan; ^2^ Division of Reconstructive Surgery for Oral and Maxillofacial Region Department of Tissue Regeneration and Reconstruction Niigata University Graduate School of Medical and Dental Sciences Niigata Japan; ^3^ Department of Oral and Maxillofacial Surgery Nagoya University Graduate School of Medicine Nagoya Japan

**Keywords:** MEDICATION‐RELATED OSTEONECROSIS OF THE JAW, SECRETOME, CYTOKINE, OSTEOGENESIS, CELL MIGRATION

## Abstract

Recently, several studies have demonstrated that intravenous administration of mesenchymal stem cells (MSCs) improve medication‐related osteonecrosis of the jaw (MRONJ), and paracrine effects of secretomes from MSCs have been hypothesized as the primary contributors. These secretomes in conditioned media from human MSCs (MSC‐CM) were previously demonstrated to promote bone and tissue regeneration. Because MSC‐CM contain cytokines monocyte chemoattractant protein (MCP)‐1, insulin growth factor (IGF)‐1, and vascular endothelial growth factor (VEGF) at relatively higher concentrations than other factors, these cytokines were considered as relevant active factors for tissue regeneration. By mixing the recombinant proteins of MCP‐1, IGF‐1, and VEGF, included at the same concentrations in MSC‐CM, we prepared cytokine mixtures mimicking MSC‐CM and then evaluated its therapeutic effects in a rat MRONJ model. In vitro, cytokine mixtures promoted osteogenic differentiation, migration, and proliferation of rat MSCs. In addition, these maintained osteoclastic function. In vivo, we used a rat MRONJ model to examine therapeutic effects of the cytokine mixtures through intravenous administration. In MSC‐CM or cytokine mixture group, open alveolar sockets in 66% or 67% of the rats with MRONJ, respectively, healed with complete soft tissue coverage and socket bones, whereas in the other groups, the exposed necrotic bone with inflamed soft tissue remained. Histological analysis revealed new bone formation and the appearance of osteoclasts in MSC‐CM or cytokine mixture group; however, osteoclasts were significantly reduced in the other groups. Thus, we concluded that intravenous administration of cytokine mixtures might be an effective therapeutic modality for treating patients with MRONJ. © 2017 The Authors *JBMR Plus* published by Wiley Periodicals, Inc. on behalf of American Society for Bone and Mineral Research

## Introduction

Bisphosphonates (BPs) were originally developed as drugs for the treatment of bone‐resorbing diseases such as multiple myeloma and bone metastasis, whose typical origins are breast and prostate cancers as well as tumor‐related hypercalcemia,^1^, ^2^, ^3^, ^4^ and for preventing pathological fractures in patients with osteoporosis. Lately, several reports have suggested that a rare but potentially severe side effect of BPs is medication‐related osteonecrosis of the jaw (MRONJ),^5^ defined as an exposed bone in the maxillofacial region that persists for >8 weeks in patients with present or previous BP treatment without a history of radiation therapy in the jaw.

To date, various attempts to control this disorder have been unsuccessful, and standard osseous sequestrectomy usually results in further enlargement of the bone defects.^6^, ^7^ Therefore, conservative, nonsurgical approaches have been recommended for the management of MRONJ, which slow its deterioration but do not cure the disease.^8^, ^9^, ^10^ Thus, development of an effective approach is an urgent concern for the prevention and treatment of patients with MRONJ.

Bone marrow‐derived mesenchymal stem cells (MSCs) have been extensively investigated in clinical practice as tools for the regeneration of bones.^11^, ^12^ In fact, some studies have reported that an intravenous injection of MSCs improves MRONJ.^13^, ^14^ However, several issues with stem cells remain to be addressed, including tumorigenesis,^15^ poor survival of implanted cells,^16^ and transmission of infectious diseases. Because implanted MSCs may contribute to tissue regeneration not only via pluripotency but also via paracrine effects,^17^, ^18^ secretomes in conditioned media from human MSCs (MSC‐CM), which are known to include various types of cytokines and other factors, may help address these issues.

We previously reported therapeutic effects of MSC‐CM in a rat MRONJ model.^19^ Furthermore, we reported that MSC‐CM contains numerous cytokines, including monocyte chemoattractant protein (MCP)‐1, insulin growth factor (IGF)‐1, and vascular endothelial growth factor (VEGF).^(19,20)^ These cytokines regulate migration, angiogenesis, antiapoptosis, and osteogenesis in host MSCs, osteoclast precursors, or immune cells in inflamed tissues and may thus accelerate the regeneration of bone and other tissues.^21^, ^22^

It is possible that only a small number of specific factors in MSC‐CM are capable of regenerating bone and other tissues. In fact, we hypothesized that MCP‐1, IGF‐1, and VEGF are the relevant active factors because according to our previous reports,^20^ these were present in MSC‐CM at relatively higher concentrations than the other factors and are well known to affect cellular migration, angiogenesis, antiapoptosis, and osteogenesis. In the present study, these cytokines (MCP‐1, IGF‐1, and VEGF) were mixed at the concentrations detected in MSC‐CM, and the mixtures were prepared to mimic MSC‐CM. Therapeutic effects of these cytokine mixtures on MRONJ was evaluated and compared with those of MSC‐CM by using a rat MRONJ model.

## Materials and Methods

### Animal experiments

All animal experiments conducted in this study were in strict accordance with the protocols approved by the Guidelines for Animal Experimentation of the Nagoya University School of Medicine (approval nos. 25374 and 26063).

### Cell preparation

Human MSCs (hMSCs) were purchased from Lonza Inc. (Walkersville, MD, USA) and cultured in MSC basal medium (Lonza Inc.) containing MSCGM SingleQuots (Lonza Inc.) at 37°C in 5% CO_2_/95% air. After primary culture, the cells were subcultured at a density of approximately 1 × 10^4^ cells/cm^2^. For the experiments, hMSCs at third to sixth passages were used.

Rat MSCs (rMSCs) were isolated from 7‐week‐old Wistar/ST male rats (Japan SLC, Shizuoka, Japan) as previously reported.^(23)^ In brief, donor rats were euthanized and the femora were dissected out. Under sterile conditions, the edge of each bone was cut, Dulbecco's modified Eagle medium (DMEM; Gibco, Rockville, MD, USA) was injected into the bone marrow using an 18‐gauge syringe, and bone marrow cells were flushed out to the opposite side; this procedure was repeated several times. The marrow was then seeded into each tissue culture flask in DMEM containing an antibiotic–antimycotic solution (100 units/mL penicillin G, 100 mg/mL streptomycin, and 0.25 mg/mL amphotericin B; Gibco), and the medium was supplemented with 10% fetal bovine serum (FBS). Three days after seeding, the floating cells were removed and the medium was replaced with a fresh medium. The adherent, spindle‐shaped cells were passaged when the cells approached confluence. The adherent cells were collected using trypsin/EDTA, resuspended in fresh medium, and transferred to new flasks at a density of 1 × 10^4^ cells/cm^2^.

After zoledronate (Zol) (35 µg/kg/wk) (Zometa, Novartis Pharmaceuticals, Tokyo, Japan) and dexamethasone (Dex) (1 mg/kg/d) (Decadron, Merck & Co. Inc., Tokyo, Japan) were subcutaneously injected into 5‐week‐old Wistar/ST male rats for 2 weeks,^23^, ^24^ rMSCs were isolated using the aforementioned method (Zol rMSCs). The pluripotency of cells obtained for differentiation into classic mesenchymal lineage cells, including osteoblasts, adipocytes, and chondrocytes, was verified using previously reported methods.^25^ These cells were used in this study as rMSCs or Zol rMSCs.

Rat osteoclast precursors were purchased from Cosmo Bio Company, Limited (Tokyo, Japan) and cultured in basal medium (BM; α‐minimum essential medium [α‐MEM], FBS, 50 ng/mL macrophage colony‐stimulating factor [M‐CSF], and 15 ng/mL receptor activator of NF‐κB ligand [RANKL]) at 37°C in 5% CO_2_/95% air.

### Preparation of conditioned media

Eighty‐percent confluent hMSCs were replenished with serum‐free DMEM (Gibco) containing antibiotic–antimycotic solution. The cell‐cultured conditioned media (CM) were collected after 48‐hour incubation. Subsequently, the CM were centrifuged at 440*g* for 5 minutes at 4°C. The supernatant was collected and centrifuged at 17,400*g* for 3 minutes at 4°C, filtered at 0.22 µm (Millex‐GP; Merck Millipore Ltd., Billerica, MA, USA), and used as MSC‐CM. MSC‐CM was stored at −80°C before being used for the experiments detailed below.

### Cytokine mixtures

Recombinant human MCP‐1 (PeproTech, Rocky Hill, NJ, USA), recombinant human IGF‐1 (Somazon; Astellas Pharma Inc., Tokyo, Japan), and recombinant human VEGF (Wako Pure Chemical Industries Ltd., Osaka, Japan) were prepared at concentrations of 1100, 1500, and 770 pg/mL to match the corresponding concentrations in MSC‐CM.^19^ We designated cytokine mixtures of MCP‐1, IGF‐1, and VEGF as MIV.

### Cytokine depletion assay

Cytokines were depleted from the CM using rabbit antihuman polyclonal antibodies against IGF‐1 (LS‐C36891; LifeSpan BioSciences Inc., Seattle, WA, USA), VEGF (ab39250; Abcam, Cambridge, UK), and MCP‐1 (ab25124; Abcam). In brief, Protein G Mag Sepharose (GE Healthcare Ltd., Little Chalfont, UK) was precoated with 30 ng/mL anti‐IGF‐1 and gently mixed with CM overnight at 4°C using an MTR*‐*103 rotator (AS ONE Co., Osaka, Japan). Beads were then removed by centrifugation for 10 minutes at 1500*g* and the supernatant was depleted in a similar manner using 15 ng/mL anti‐VEGF and 30 ng/mL anti‐MCP‐1. Enzyme‐linked immunosorbent assay (ELISA) was used to confirm depletion. Accordingly, we designated MSC‐CM‐depleted MCP‐1, IGF‐1, and VEGF as MSC‐CM‐dep MIV.

### Cell viability assay

BPs are strong inhibitors of MSCs or mature osteoclast functions, including bone resorption and survival.^26^, ^27^ To test whether Zol‐induced inhibition in rMSCs and rat osteoclasts (rOCs) was improved by MIV, we performed an 3‐(4,5‐dimethylthiazol‐2‐yl)‐2,5‐diphenyltetrazolium bromide (MTT) assay.

rMSCs and rOCs (Cosmo Bio Co. Ltd., Tokyo, Japan) were cultured at a density of 1 × 10^4^ and 3 × 10^4^ cells/well, respectively, in 96‐well culture plates at 37°C in 5% CO_2_/95% air. When the cells became 80% confluent, these were treated with concentrations of 150 µM Zol (clinical concentrations) with or without MSC‐CM or cytokine mixtures for 48 hours. After the indicated culture time, MTT assay was performed by incubation of cells with 0.2% MTT solution (Cell Counting Kit, DOJINDO, Tokyo, Japan) at 37°C for 4 hours. The resulting color was then analyzed by measuring the absorbance at 450 nm (A450), wherein A450 corresponded to the viability of the cells.^27^

### Pit formation assay

To confirm the function of rOCs, we performed a pit formation assay. rOC precursors were cultured at a density of 6 × 10^4^ cells/well in a 96‐well Osseo Assay Stripwell Plate (Corning Inc., Corning, NY, USA) at 37°C in 5% CO_2_/95% air for 10 days. These were treated with BM, BM + 150 µM Zol, BM + 150 µM Zol + MSC‐CM, BM + 150 µM Zol + MIV, or BM + 150 µM Zol + MSC‐CM‐dep MIV for 10 days. Six wells were used per group. To measure the areas containing resorption, we performed the von Kossa staining. Cells in the well plates were fixed using 100% ethanol and then stained with 5% silver nitrate, placed under a UV lamp for 30 minutes, and rinsed with distilled water before treatment with 5% sodium thiosulfate for 2 to 3 minutes. Images of the resorbed area were measured using light microscopy and results were expressed as the total area resorbed in at least six random fields, as evaluated using Image J software (version 1.67; NIH, Bethesda, MD, USA).

### Alizarin red staining

For mineralization analysis, Zol rMSCs were cultured with continuous exposure to osteogenic induction medium (OM; Osteogenic Differentiation Medium BulletKit; Lonza Inc.) with MSC‐CM, MIV, or MSC‐CM‐dep MIV for 14 days and were subsequently fixed using 90% ice‐cold ethanol for 10 minutes. After fixation, ethanol was removed and the amount of mineralization was measured using 1% alizarin red S (pH 6.2) for 30 minutes at RT. The stained cultures were subsequently measured for A570 and photographed.^27^

### Wound healing assay

The migratory properties of Zol rMSCs were examined using the CytoSelect Wound Healing Assay kit (Cell Biolabs, San Diego, CA, USA) according to the manufacturer's instructions. In brief, cell suspension was added to the well using a plastic insert. The insert was removed from the well after a monolayer of cells had formed, creating a wound gap of 0.9 mm. After washing, the cells were incubated at 37°C for 48 hours with MSC‐CM, MIV, MSC‐CM‐dep MIV, 30% FBS, or serum‐free DMEM (DMEM [−]). The extent of wound closure was determined using a light microscope (CK40; Olympus, Tokyo, Japan).

### Real‐time reverse transcription polymerase chain reaction (RT‐PCR) analysis

Zol rMSCs were cultured with DMEM (−), MSC‐CM, MIV, or MSC‐CM‐dep MIV for 48 hours. Total RNA was extracted using the RNeasy Mini Kit (Qiagen GmbH, Hilden, Germany) according to the manufacturer's instructions. RT‐PCR analysis was performed as previously described.^28^, ^29^ The sequence of specific primers and probes used for real‐time RT‐PCR analysis of alkaline phosphatase (*alp*), runt‐related transcription factor 2 (*runx2*), *vegf*, *rankl*, osteoprotegerin (*opg*), and *gapdh* are listed in Table 1.

**Table 1:** Primer Sequences Used for Real‐Time RT‐PCR

Gene	Sequence	Accession no.
*alp*		
F	GACAGTCATTGAATACAAAAC	NM_013059
R	ACGGAATTCTTGGTTAGTA	
Probe	TAAGCCATCTCGCCTGCCAT	
*runx2*		
F	CCTCTTATCTGAGCCAGA	NM_053470
R	GCAGTGTCATCATCTGAA	
Probe	CATCCATCCATTCCACCACGC	
*vegf*		
F	ATCCCGGTTTAAATCCTG	NM_031836
R	GGAACATTTACACGTCTG	
Probe	CACTGTGAGCCTTGTTCAGAGC	
*rankl*		
F	GTCGTTAAAACCAGCATC	NM_057149
R	CCTGACCAGTTCTTAGTG	
Probe	TCCCAAGTTCGCATAACCTGATGA	
*opg*		
F	CGAAGAGGCATTCTTCAG	NM_012870
R	TCTGCATTCACTTTGGTC	
Probe	TGCTGTGCCTACCAAGATTATACCG	
*gapdh*		
F	GTTCCAGTATGACTCTACC	NM_017008
R	TCACCCCATTTGATGTTA	
Probe	TTCAACGGCACAGTCAAGGC	

F = forward primer; R = reverse primer; probe = Taqman probe; *alp =* alkaline phosphatase; *runx2 *= runt‐related transcription factor 2; *vegf =* vascular endothelial growth factor*; rankl = *receptor activator of NF‐κB ligand; *opg =* osteoprotegerin.

RT‐PCR and the resulting relative increase in reporter fluorescent dye emission were monitored in real time using the 7000 Sequence Detector (Perkin‐Elmer, Foster City, CA, USA). Signals were analyzed using Sequence Detector Software (version 1.1; Perkin‐Elmer). The PCR conditions were as follows: one cycle at 50°C for 2 minutes, one cycle at 60°C for 30 minutes, one cycle at 95°C for 5 minutes, 50 cycles at 95°C for 20 seconds (denaturation), and then 60°C for 1 minute (annealing and extension). The relative amount of each mRNA in one sample was obtained by calculating the respective standard curves. The standard curves for each mRNA were drawn using different concentrations (625, 125, 25, 5, and 1 ng) of total RNA of rMSCs. The relative expression levels were normalized to *gapdh* expression.

### Western blot analysis

Immunoblot analysis was performed as previously described.^30^ In brief, the cultured cells were lysed in a buffer containing 20 mM Tris (pH 7.4), 150 mM NaCl, 1 mM EDTA, 1 mM EGTA, 1% Triton X‐100, 25 mM sodium pyrophosphate, 1 mM NaF, 1 mM β‐glycerophosphate, 0.1 mM sodium orthovanadate, 1 mM phenylmethyl sulfonyl fluoride, 2 µg/mL leupeptin, and 10 µg/mL aprotinin. Overall, 50 µg of the total cell lysate were separated on SDS‐PAGE gels and transferred onto polyvinylidene difluoride (PVDF) membranes (Millipore). The membranes were blocked using 5% nonfat dry milk in tris‐buffered saline with Tween‐20 (TBS‐T) buffer and incubated with the following primary antibodies: ALP, 1:1000 (ab95462; Abcam); RUNX2, 1:1000 (ab23981; Abcam); VEGF, 1:1000 (ab46154; Abcam); mammalian target of rapamycin (m‐TOR), 1:1000 (ab2732; Abcam); and β‐actin, 1:1000 (ab8227; Abcam) at 4 °C overnight. The samples were then incubated for 1 hour at RT with horseradish peroxidase (HRP)‐conjugated anti‐rabbit secondary antibody (1:2000; Abcam). Signals were detected using the ECL Western Blotting Kit (Amersham Pharmacia Biotech, Piscataway, NJ, USA). For quantification, density of the bands was measured using Image J software (verion 1.67; NIH).

### Generation of a rat MRONJ model and therapeutic effects of MIV

We evaluated a rat MRONJ model in our previously report^19^ and established that model. In brief, 5‐week‐old Wistar/ST male rats received Zol (35 µg/kg/wk) and Dex (1 mg/kg/d) subcutaneously for 2 weeks.^19^, ^31^ Thereafter, unilateral maxillary molars were extracted with minimal trauma to the surrounding tissues under deep anesthesia using intraperitoneal injection of ketamine (60–90 mg/kg) and xylazine (100–150 mg/kg). We defined only the rats with bone exposure as the MRONJ model. Two weeks after tooth extraction, we defined the following groups based on the material used for the intravenous injection: 1) nontreatment group, not administered; 2) DMEM (−) group (1 mL), DMEM (−) administered; 3) MSC‐CM group (1 mL), MSC‐CM administered; 4) MIV group (1 mL), MIV administered; and 5) MSC‐CM‐dep MIV group (1 mL), MSC‐CM‐dep MIV administered. The rats were euthanized at 2 weeks after intravenous injection (*n* = 8 per group).

### Radiographic and histological analyses

Surgical sites were dissected, fixed in 4% PFA, and subjected to micro‐computed tomography (micro‐CT) analysis using a laboratory X‐ray CT device (SkyScan1176; Bruker, Kontich, Belgium). Using the software supplied with the instrument, two‐dimensional (2D) and three‐dimensional (3D) images were reconstituted.

After the radiological assessment, explants were decalcified using K‐CX solution (Falma Co., Tokyo, Japan), dehydrated with graded ethanol, cleared with xylene, and embedded in paraffin. The specimens were sagittally cut to create 5‐µm‐thick histological sections and stained using hematoxylin and eosin (H & E).

Empty osteocytic lacunae were counted in at least 3 random fields and expressed as a percentage of total bone lacunae. Osteoclasts were identified using tartrate‐resistant acid phosphatase (TRAP) staining with the leukocyte acid phosphatase kit (Sigma‐Aldrich, Tokyo, Japan) according to the manufacturer's instructions. Histological analysis was performed using light microscopy.

### Immunohistochemistry

Fresh frozen samples were sectioned at 8 µm according to the Kawamoto method using the Multi‐Purpose Cryosection Preparation Kit (Leica Microsystems, Tokyo, Japan).^32^ Each section was fixed in 100% ethanol for 10 minutes at RT, washed, blocked with 5% bovine serum albumin/PBS for 30 minutes, and stained with primary antibodies in blocking buffer overnight at 4°C. The sections were then stained with secondary antibodies for 30 minutes, washed, stained with 4′,6‐diamidino‐2‐phenylindole (DAPI; Invitrogen, Tokyo, Japan) for 15 minutes, mounted with SCMM R3 (Leica), and examined using fluorescence microscopy (BZ‐9000, Keyence, Osaka, Japan). Purified mouse antibody against rat CD31 (Purified Mouse Anti‐Rat CD31; BD Pharmingen, Tokyo, Japan) was used for immunostaining. Secondary antibodies were conjugated using Alexa Fluor 633 (Invitrogen).

### Statistical analysis

All experiments were performed in triplicate and repeated at least twice. Group means and standard deviations (SDs) were calculated for each measured parameter. Statistical differences were evaluated using Tukey's honestly significant difference test. A *p* value <0.05 was considered statistically significant and that <0.01 was considered statistically very significant.

## Results

### Effect of cytokine mixtures on rMSCs or rOCs viability

**Figure 1:**
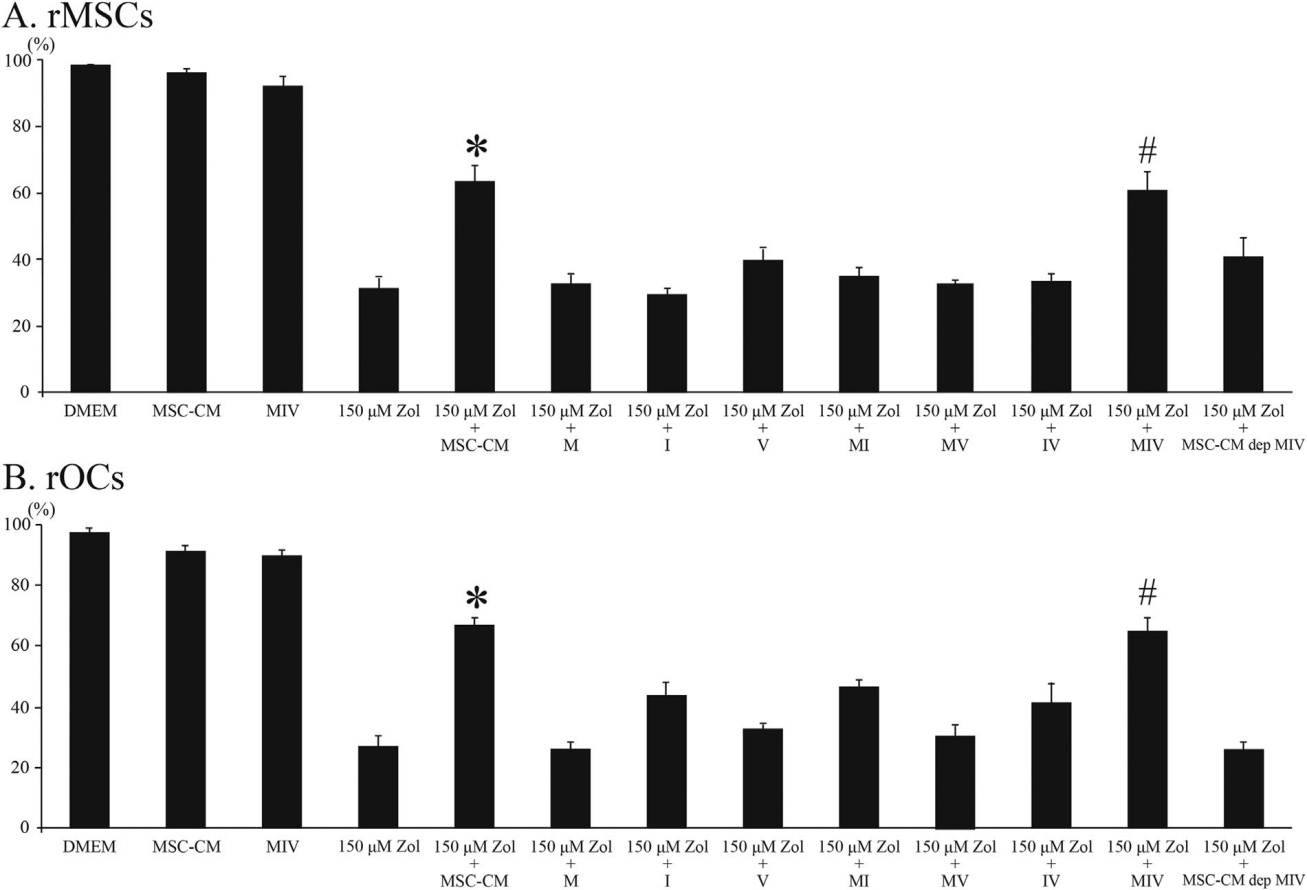
The effects of Zol, MSC‐CM, and MIV on viability of rMSCs and rOCs. (*A*, *B*) With the addition of Zol at 150 µM, viability of rMSCs and rOCs decreased. However, with the addition of Zol at 150 µM with MSC‐CM or MIV, this viability improved. Thus, MIV, the three‐cytokine mixture, enhanced the proliferation of rMSCs and rOCs. Data are presented as mean ± SD of three independent experiments. *p < 0.05 versus others; ^#^ *p* < 0.05 versus others.

MTT assay was performed to examine its effect on cytotoxicity and cell viability of rMSCs and rOCs. The results in Fig. 1 *A*, *B* indicate that cell viability was significantly increased with the addition of 150‐µM Zol‐added MSC‐CM or MIV. However, the viability of rMSCs and rOCs was decreased with only Zol, single cytokine, two combination cytokines, or MSC‐CM‐dep MIV. Based on these results, three‐cytokine mixtures, mainly MIV, may contribute to cell viability.

### MIV maintains resorptive activity of osteoclasts

**Figure 2:**
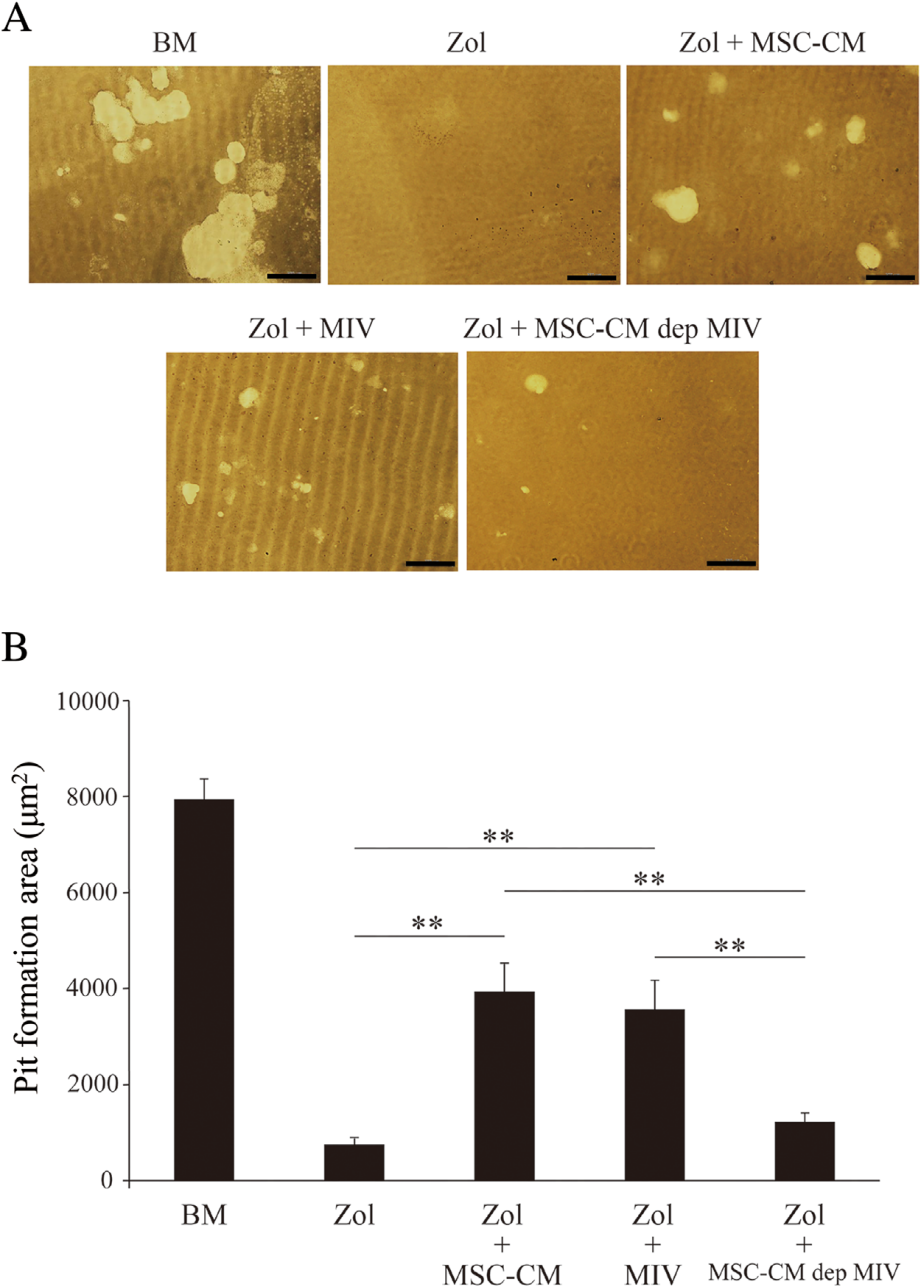
MIV maintains osteoclastic function. (*A*) Photomicrographs of the resorbed area (white areas) under each condition. (*B*) Images of the resorbed area were measured using Image J software. The results are expressed as total area resorbed in at least three random fields. Scale bars = 200 µm. Data are presented as mean ± SD. **p < 0.01.

A pit formation assay was then performed to test the resorptive activity of osteoclasts (Fig. 2 *A*). A function maintenance effect on pit number and area was observed on the Osseo Assay Stripwell Plate (Corning Inc.) in Zol + MSC‐CM or Zol + MIV group, whereas an inhibitory effect was observed in Zol or Zol + MSC‐CM‐dep MIV group. Fig. 2 *B* shows quantification of the resorptive areas. Thus, MIV showed function maintenance in osteoclasts.

### MIV promotes osteogenic differentiation in Zol rMSCs

**Figure 3:**
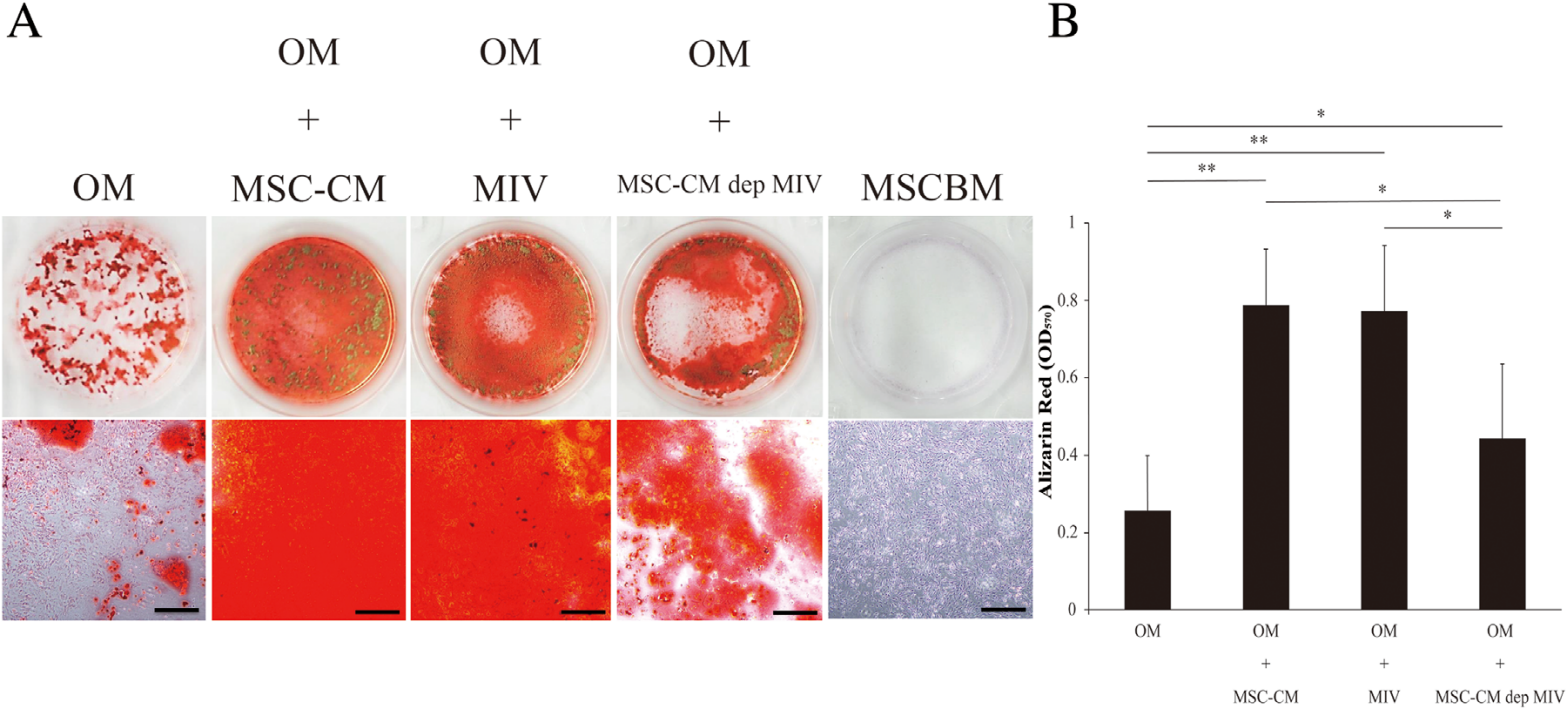
The effects of MIV on osteogenic differentiation. Zol rMSCs were cultured with continuous exposure to OM with MSC‐CM, MIV, or MSC‐CM‐dep MIV. (*A*) After 14 days, photographs reveal staining with 1% alizarin red S, and bar chart shows measured absorbance. Zol rMSCs + OM exhibited weak alizarin red staining, Zol rMSCs + OM + MSC‐CM or MIV exhibited strong alizarin red staining, and Zol rMSCs + OM + MSC‐CM‐dep MIV exhibited weak alizarin red staining. Scale bars = 200 µm. (*B*) The stained cultures were then measured for absorbance at 570 nm and quantified. Data are presented as mean ± SD of three independent experiments. **p < 0.01; *p < 0.05.

The osteogenic differentiation potential of Zol rMSCs was assessed using alizarin red staining. When treated with OM, Zol rMSCs exhibited weak alizarin red staining. However, when treated with MSC‐CM or MIV together with OM, Zol rMSCs exhibited strong alizarin red staining (Fig. 3 *A*, *B*) and when treated with MSC‐CM‐dep MIV together with OM, these exhibited weak alizarin red staining (Fig. 3 *A*, *B*). Based on these results, primarily MIV participated in the osteogenic differentiation of Zol rMSCs.

### Effects of MIV on the migration of Zol rMSCs

**Figure 4:**
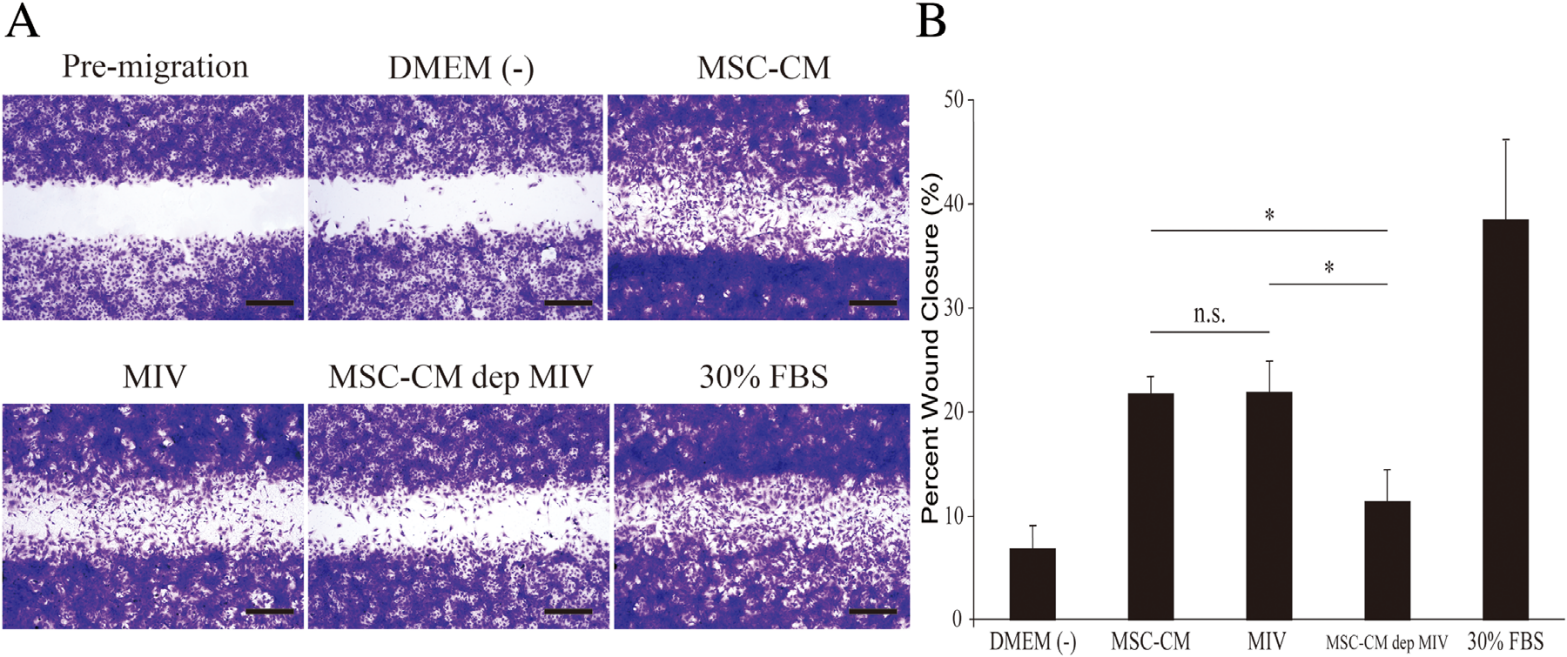
MIV promoted migration and proliferation of Zol rMSCs in the wound healing assay. (*A*) Migration of Zol rMSCs. Wounds were generated as described in Materials and Methods (scale bars = 500 µm). (*B*) The level of cellular fill within the wound area in response to MSC‐CM or MIV groups was compared with the wound‐fill response in the presence of MSC‐CM‐dep MIV. Data are presented as mean ± SD of five independent experiments. *p < 0.05.

The proportion of Zol rMSCs in the wound area of DMEM (−) was 6.91% ± 2.11%. There were 38.6% ± 7.46% Zol rMSCs in the wound area of positive controls (30% FBS). MSC‐CM and MIV induced significant effects (*p* < 0.05) and closed the wound to 21.9% ± 1.52% and 22.0% ± 2.88% Zol rMSCs, respectively (Fig. 4 *A*, *B*). However, compared with MSC‐CM and MIV, MSC‐CM‐dep MIV did not induce any significant effects and did not close the wound. Thus, compared with DMEM (−), MIV increased the migration of Zol rMSC more than threefold. These differences were statistically significant (*p* < 0.05), which indicates that MIV enhanced the migration of Zol rMSC (Fig. 4 *A*, *B*).

### MIV enhances osteogenic and angiogenesis gene markers or proteins and also enhances the central regulator of migration

**Figure 5:**
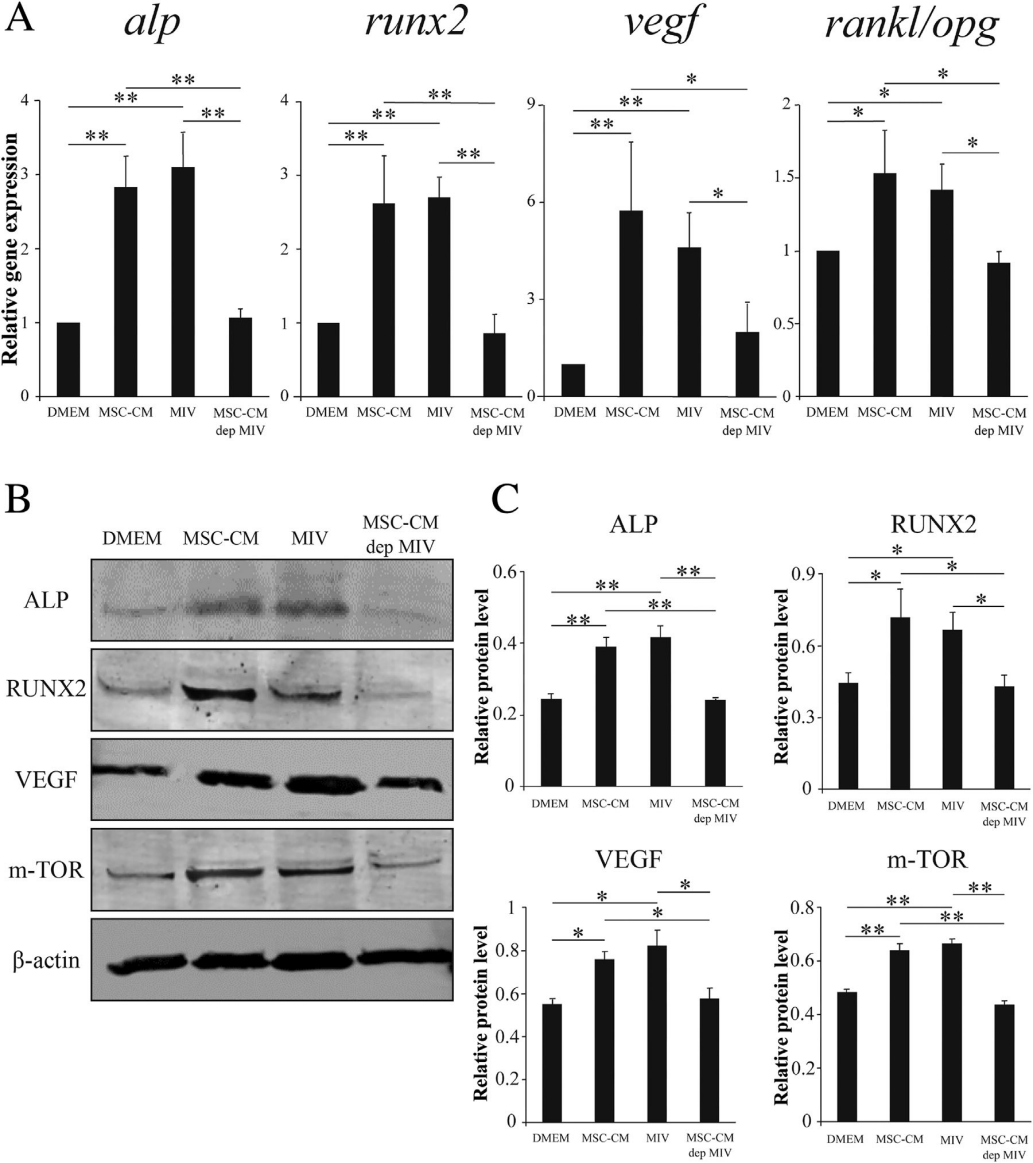
The results of real‐time RT‐PCR or Western blot analysis. (*A*) The levels of expression of *alp*, *runx2*, and *vegf* genes and the *rankl/opg* ratio were significantly upregulated in MSC‐CM and MIV groups. (*B*) The detection of ALP, RUNX2, VEGF, and m‐TOR protein expression levels was conducted using Western blot analysis. β‐actin was used as the internal control. Osteogenic, angiogenic, and migration‐related proteins were significantly increased in MSC‐CM and MIV groups. (*C*) For quantification, density of the bands was measured using Image J software. Data are presented as mean ± SD of three independent experiments. **p < 0.01; *p < 0.05.

To investigate the effects of MIV on Zol rMSCs, we performed RT‐PCR and Western blot analyses. The expression levels of *alp*, *runx2*, and *vegf* genes and the *runkl/opg* ratio were significantly upregulated in Zol rMSCs cultured in MIV compared with those cultured in DMEM(−) or MSC‐CM‐dep MIV (Fig. 5 *A*). Of note, mRNA expressions of these four genes were similar in MIV and MSC‐CM (Fig. 5 *A*).

Next, we examined the effects of MIV on ALP, RUNX2, VEGF, and m‐TOR (a central regulator of proliferation, migration, and survival^33^) in Zol rMSCs using Western blot analysis. In addition to mRNA levels, protein levels in Zol rMSCs also confirmed that ALP, RUNX2, VEGF, and m‐TOR levels were higher in the MIV group than those in the MSC‐CM‐dep MIV group. However, the protein levels in the MIV group were the same as those in the MSC‐CM group (Fig. 5 *B*, *C*). These results suggest that MIV upregulates osteogenesis, angiogenesis, and migration.

### MIV injection cured MRONJ in rats as same as MSC‐CM injection

**Figure 6:**
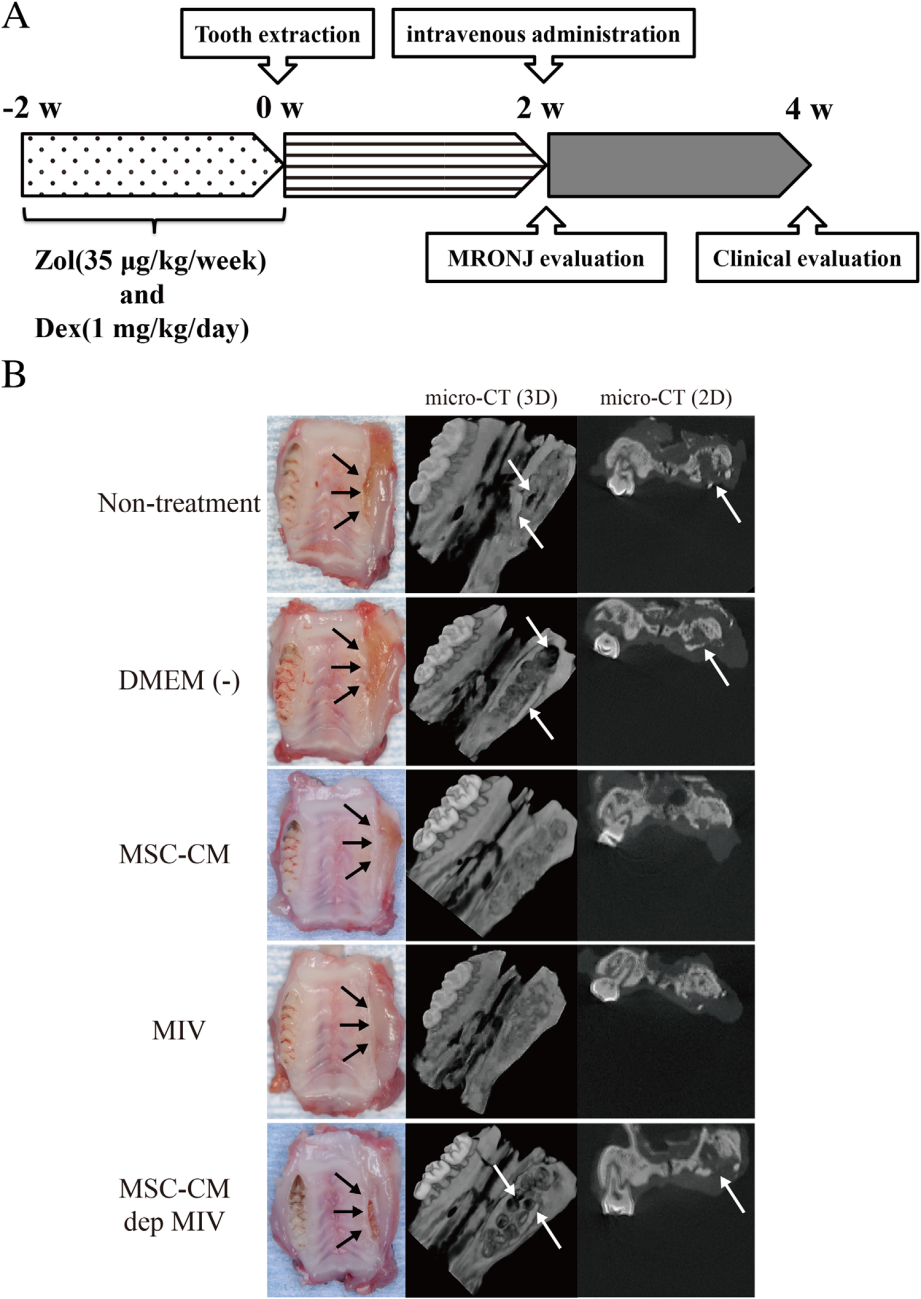
Therapeutic effects of MIV in a rat MRONJ model. (*A*) Protocols for the generation of MRONJ and experimental design. (*B*) Representative gross clinical appearance of gingival mucosa and necrotic bone at the extraction sites 2 weeks after each injection and micro‐CT analysis. Necrotic bone (white arrow) and unhealed open sockets were recognized. However, in the MSC‐CM or MIV groups, healed gingival mucosa and healed bone sockets were recognized.

An equivalent treatment protocol of high‐dose intravenous nitrogen‐containing BPs, Zol, and an immunosuppressive drug, Dex, is required for the development of a MRONJ disease model. Fig. 6 *A* describes the protocol that we previously reported.^19^ We used a rat MRONJ model revealing incomplete mucosal healing and the presence of exposed bone at 2 weeks after unilateral surgical extraction.

**Figure 7:**
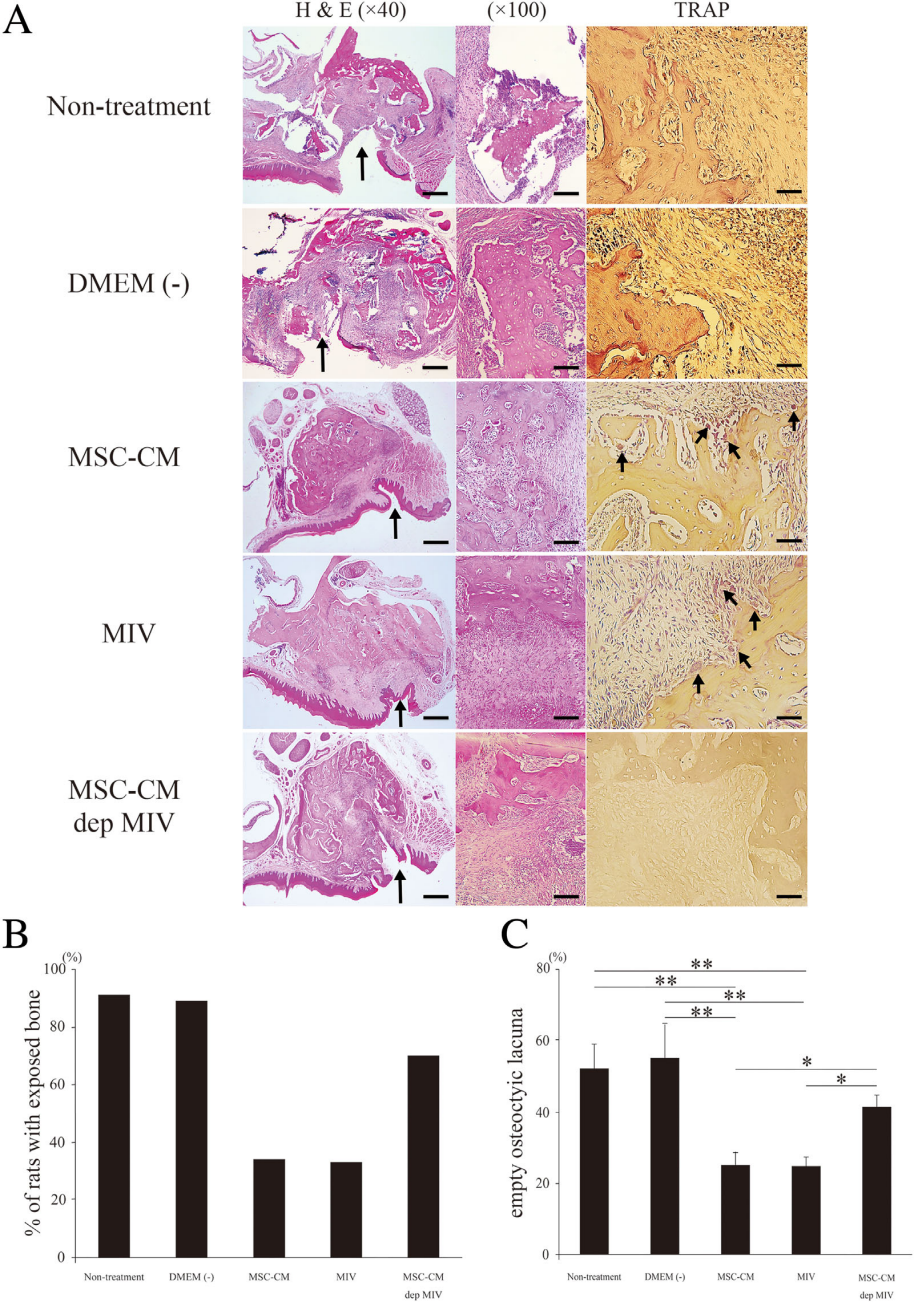
Histological images of extraction sockets. (*A*) In H&E staining, histological images of extraction sockets revealed open sockets without epithelial lining in the nontreatment, DMEM(−), and MSC‐CM‐dep MIV groups and healed gingival mucosa with complete epithelial coverage in MSC‐CM and MIV groups. In TRAP staining, in the nontreatment, DMEM(−), and MSC‐CM‐dep MIV groups, osteoclasts (black arrows) were significantly decreased compared with those in the MSC‐CM and MIV groups. H&E staining: magnification ×40, scale bars = 500 µm; ×100, scale bars = 100 µm; TRAP staining: ×400, scale bars = 50 µm. (*B*) Incidence of a BRONJ‐like lesion, manifested as an unhealed open socket with an area of exposed bone and no mucosal coverage, at the extraction site. (*C*) Empty osteocytic lacunae were counted in at least 3 random fields and expressed as a percentage of total bone lacunae. Data represent the mean ± SD. **p < 0.01; *p < 0.05.

In the MSC‐CM and MIV groups, micro‐CT examinations revealed healing of bone defects compared with that in the nontreatment, DMEM(−), and MSC‐CM‐dep MIV groups at 2 weeks after intravenous injection (Fig. 6 *B*). Two weeks after intravenous injection, open alveolar sockets of 66% or 67% of rats (*n* = 8) with MRONJ in the MSC‐CM or MIV groups, respectively, healed with complete soft tissue coverage, whereas in the nontreatment, DMEM(−), and MSC‐CM‐dep MIV groups, the exposed necrotic bone with inflamed soft tissue remained (Fig. 7 *B*). Histological analysis revealed new bone formation and the appearance of osteoclasts in the MSC‐CM and MIV groups; however, these were significantly reduced in the nontreatment, DMEM(−), and MSC‐CM‐dep MIV groups. In the MSC‐CM and MIV groups, osteoclasts were observed on socket surfaces (Fig. 7 *A*).

**Figure 8:**
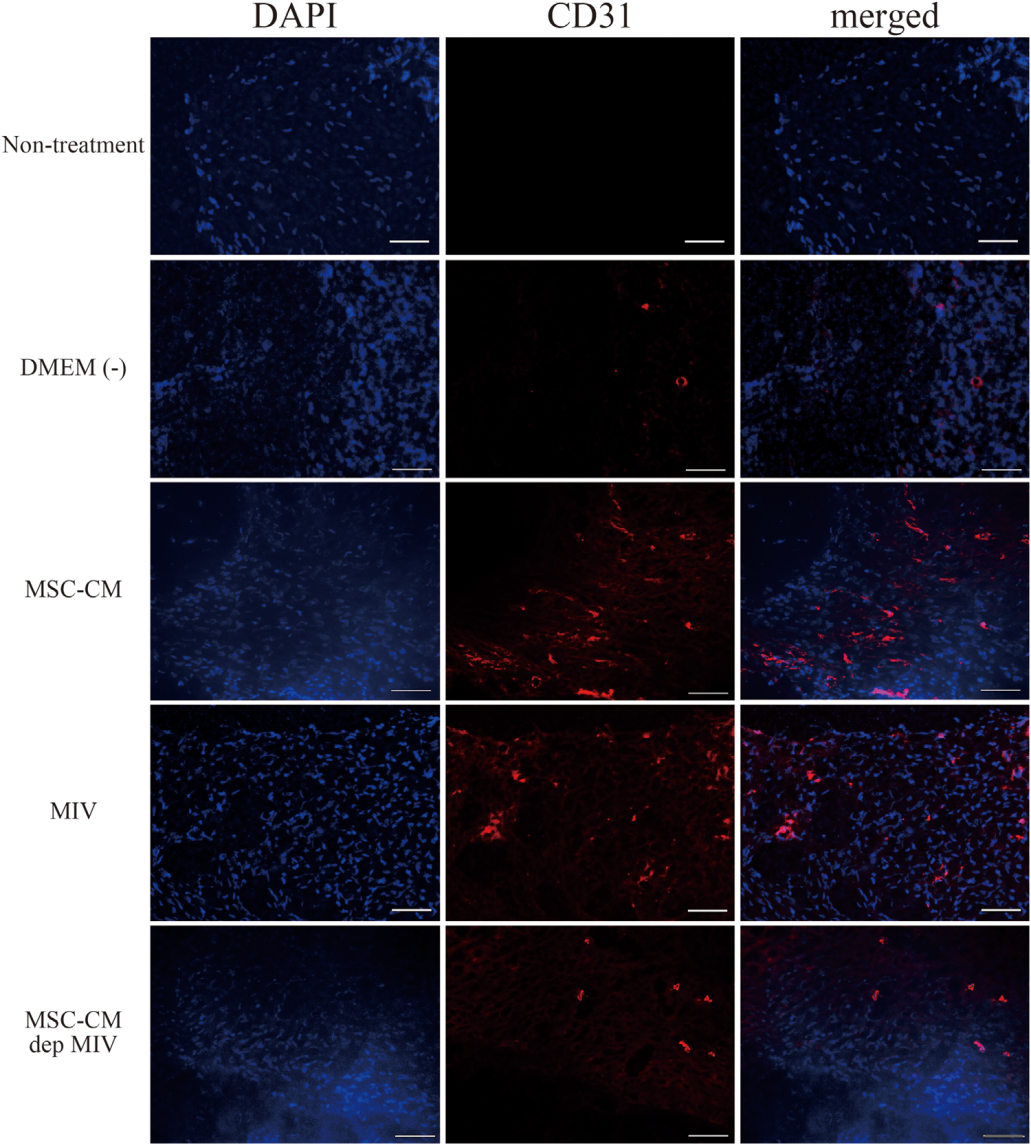
Representative images showing immunohistochemical staining of extraction sites in each group. CD31‐positive endothelial cells were increased in the MSC‐CM and MIV groups. Magnification ×400, scale bars = 50 µm.

To further characterize impaired mucosal and bone healing, we assessed blood vessels. Immunohistochemical staining against CD31 for vascular endothelial cells showed increased endothelial cells in the MSC‐CM and MIV groups compared with those in the other groups (Fig. 8). Particularly, CD31‐positive endothelial cells decreased in the MSC‐CM‐dep MIV group. Based on these results, at 2 weeks after extraction, MRONJ rats receiving MIV injections showed complete mucosal healing, bone regeneration, and angiogenesis in the extracted alveolar sockets.

## Discussion

BPs have been reported to promote osteoprotegerin, also known as osteoclastogenesis inhibitory factor, by MSCs and osteoblasts.^34^ BPs inhibit the expressions of interleukin (IL)‐6, M‐CSF, and MCP‐1 via inhibition of the Ras/MEK/ERK pathway and activation of p38MAPK in MSCs and osteoblasts,^35^ thereby inhibiting the migration and differentiation of osteoclast precursors. Another study reported that BPs suppressed mineralization through direct cytotoxicity and inhibited osteoblast differentiation^27^ or endothelial cell migration and differentiation.

Therapeutic effects of MSCs in tissue engineering and regenerative medicine can be partly attributed to paracrine pathways^18^, ^24^ triggered by several factors secreted into the CM. In fact, we have reported that MSC‐CM contains numerous cytokines^20^ such as MCP‐1, IGF‐1, and VEGF, which can synergistically affect migration, osteogenesis, and angiogenesis. The effects of these three cytokines are thought to be very complex, although IGF‐1 is believed to regulate the migration of osteoblasts and MSCs,^36^ and sustained systemic or local infusion of IGF‐1 was shown to enhance bone formation.^37^ On the other hand, VEGF is a master regulator of angiogenesis and enhances survival and differentiation in endothelial cells that promote osteogenesis,^38^ and MCP‐1 is a chemokine that recruits stem cells and immune cells to inflamed tissues.^39^

MSC‐CM contains numerous other soluble factors that may positively and negatively regulate bone and tissue regenerations; hence, it is necessary to identify the specific factors involved in bone and soft tissue regenerations. In this study, we hypothesized MCP‐1, IGF‐1, and VEGF as the relevant factors, and accordingly evaluated the activity and pharmacological effects of a defined mixture of these cytokines as well as those of MSC‐CM from which these cytokines had been depleted. Concentrations of these MCP‐1, IGF‐1, and VEGF in MSC‐CM, measured using ELISA, were 1103.96 ± 395.50, 1515.60 ± 211.83, and 772.55 ± 261.82 pg/mL, respectively; the cytokine mixture was prepared using 1100 pg/mL MCP‐1, 1500 pg/mL IGF‐1, and 770 pg/mL VEGF to match these concentrations.

Surprisingly, our data indicated that similar to MSC‐CM, a defined mixture of cytokines enhanced the migration (Fig. 4) and expression of osteogenic and angiogenic marker genes in Zol rMSCs in vitro (Fig. 5) and had antiapoptotic effects on osteoclast precursors (Fig. 2). The clinically obvious MRONJ, specifically, open sockets with exposed necrotic bone and no mucosal lining, persisted beyond 2 to 3 weeks, and normal course of healing was observed in nontreated rats.^19^ In our rat MRONJ model, MIV injection improved MRONJ in a similar manner as MSC‐CM injection, but necrotic bone was not removed and sockets remained open in the other groups (Figs. 6 and 7).

Studies have also reported cooperative effects of these cytokines, which they exert on each other: IGF‐1 upregulates VEGF expression through hypoxia‐inducible factor‐2a.^40^ In addition, Chen and colleagues reported that MSCs could secrete microvesicles that contain various factors contributing to their angiogenesis‐promoting function; among these, VEGF and MCP‐1 might be of greater importance than the other cytokines.^41^ Moreover, Lu and colleagues reported that MCP‐1 is a major ligand of CC chemokine receptor 2 (CCR2) to recruit monocytes and stem cells into injured tissues in order to perform phagocytosis and produce IGF‐1 for injury repair.^42^ In our previous study that used a rat calvarial bone defect model, we demonstrated that three‐cytokine mixture promotes the migration of stem cells and osteogenesis compared with that by a single cytokine or two‐cytokine mixture.^43^ Based on these findings, we assume that the cytokines in the three‐cytokine mixtures may effectively activate multiple stages of the regeneration of bone and soft tissue, individually or in cooperation with each other.

In summary, this is the first study to report defined cytokine mixtures mimicking MSC‐CM as a potential treatment for MRONJ. Such defined cytokine mixtures can be used as standardized formulations; however, the standardization of cells or cell‐derived products from hMSCs can be challenging. Moreover, those can be directly used during surgery without any need to preculture patient MSCs. Intravenous administration of MIV provides an effective therapeutic modality for treating patients with MRONJ, which also provides insights regarding potential clinical applications.

## Disclosures

All authors state that they have no conflicts of interest.
